# Effect of Alcohol on Diffuse Axonal Injury in Rat Brainstem: Diffusion Tensor Imaging and Aquaporin-4 Expression Study

**DOI:** 10.1155/2013/798261

**Published:** 2013-10-27

**Authors:** Lingmei Kong, Gengpeng Lian, Wenbin Zheng, Huimin Liu, Haidu Zhang, Ruowei Chen

**Affiliations:** ^1^Department of Radiology, The Second Affiliated Hospital, Medical College of Shantou University, Dongxia North Road, Shantou 515041, China; ^2^Department of Radiology, The First Affiliated Hospital, Medical College of Shantou University, No. 57 Changping Road, Shantou 515041, China

## Abstract

The aim of this study is to assess the effects of alcohol on traumatic brain injury by using diffusion tensor imaging (DTI) and evaluate aquaporin-4(AQP4) expression changes in rat brainstems following acute alcohol intoxication with diffuse axonal injury (DAI). We further investigated the correlation between the AQP4 expression and DTI in the brain edema. Eighty-five rats were imaged before and after injury at various stages. DTI was used to measure brainstem apparent diffusion coefficient (ADC) and fractional anisotropy (FA), with immunostaining being used to determine AQP4 expression. After acute alcoholism with DAI, ADC values of the brainstem first decreased within 6 h and then elevated. FA values began to decline by 1 h, reaching a minimum at 24 h after trauma. There was a negative correlation between ADC values and brainstem AQP4 expression at 6 h and positive correlation at 6 h to 24 h. Changes of ADC and FA values in DAI with acute alcoholism indicate the effects of ethanol on brain edema and the severity of axonal injury. The correlations between ADC values and the brainstem AQP4 expression at different time points suggest that AQP4 expression follows an adaptative profile to the severity of brain edema.

## 1. Introduction

Ethanol administration adversely affects morbidity and mortality after traumatic brain injury (TBI) by accelerating brain edema [1]. DAI plays an important role in the pathophysiology of TBI and contributes substantially to morbidity and mortality. DAI comprises primary microscopic injury of axons caused by an acceleration-deceleration injury force, the pattern of which is more accurately described as multifocal, appearing throughout the deep and subcortical white matter and being particularly common in midline structures, including the splenium of the corpus callosum and brainstem. Histopathological studies have shown that the axons are initially damaged focally, with their microstructure largely intact [2]. Several investigators have linked DAI to focal misalignments of the cytoskeletal network or changes in axolemmal permeability, depending on the severity of the injury [3].

Brain edema is a critical event in the pathophysiology of DAI caused by alcohol intoxication. Increased brain edema has been described in TBI rats receiving higher doses of alcohol as opposed to TBI rats exposed without alcohol [4]. Two major types of traumatic brain edemas are cytotoxic and vasogenic. Cytotoxic edema occurs when fluid flows from the vascular compartment, through an intact blood-brain barrier (BBB) and astrocytic foot processes, and accumulates primarily in astrocytes. In vasogenic edema, the BBB breaks down, permitting the entry of plasma fluid into extracellular spaces within the brain, leading to increased brain volume, elevated intracranial pressure, and increased extracellular space. Today the treatment of traumatic brain edema remains a therapeutic challenge, and diagnosis is still largely symptomatic. All treatment modalities presently used are directed at decreasing intracranial pressure. For example, steroids are postulated to seal the endothelial lining, thus, attenuating vasogenic brain edema formation. The prevalence of cytotoxic edema formation might explain the limited efficacy of steroids to treat traumatic brain edema [5]. Despite the clinical importance of cytotoxic brain edema and vasogenic brain edema, the molecular mechanisms of brain water accumulation and removal are still poorly understood.

AQP4, the predominant water channel in the brain, is expressed in astrocyte foot processes surrounding capillaries and the basolateral surface of ependymal cells. Astrocyte processes comprise the glial limiting membrane in both ependymal cells and subependymal astrocytes. The pattern of AQP4 expression, predominantly at the borders between the brain parenchyma and major fluid compartments, suggests involvement of AQP4 in water movement into and out of the brain parenchyma. Recent studies have shown that AQP4 could be important in the formation and resolution of brain edema. Early in cytotoxic edema, AQP4 facilitates edema fluid formation, but in vasogenic brain edema, AQP4 increases the rate of edema fluid elimination [6]. Sripathirathan et al. suggest binge ethanol-induced brain edema is potentially associated with AQP4 upregulation [7]. The expression of AQP4 after TBI is time-dependent, region-specific, and possibly implicated in the formation and resolution of TBI-induced cerebral edema [8]. 

Common imaging techniques, such as CT and conventional MRI, are poor at characterizing DAI and provide limited information on the incidence and severity of DAI. DTI is a technique that is particularly suited to the study of white matter by using a tensor model to characterize the local diffusivity of water. The two parameters measured are fractional anisotropy (FA) and the apparent diffusion coefficient (ADC). DTI parameters are now established as potential quantitative biomarkers for evaluation of the severity of axonal injury after DAI [9]. 

There are some clinical and experimental animal studies aimed at studying morbidity and mortality of alcohol on brain injury [10–12]. To our knowledge, our study is the first report using diffusion tensor imaging combined with aquaporin-4 expression to investigate the brainstem edema after DAI with acute alcohol intoxication. The findings suggest that cytotoxic and vasogenic brain edemas are two entities which can be targeted simultaneously, changes in diffusion parameters may serve as an important indicator of pathological processes, and as such diffusion-weighted MRI is important for diagnostic purposes. In this study, we hypothesized that DTI may be effective in characterizing brainstem changes after acute alcohol intoxication with DAI. We further hypothesized that astrocyte AQP4 contributes to water diffusion and changes in ADC values in rat brainstem following DAI with acute alcoholism.

## 2. Materials and Methods

### 2.1. Animal Model

All procedures were in compliance with the Shantou University Guide for Care and Use of Laboratory Animals and were approved by the Shantou University Medical College Animal Use Committee. Eighty-five adult male Sprague-Dawley rats, weighing between 250 and 300 g, were used. Rats were divided into four groups: an ethanol-plus-DAI group (AT group, *n* = 25), a DAI-only group (T group, *n* = 25), an ethanol-only group (A group, *n* = 25), and a control group (N group, *n* = 10). Animals in both the A and AT groups were administered ethanol at a dose of 15 mL/kg [13] (Hongxing Erguotou wine, 56% vol, Beijing, China) via gastric administration. In animals belonging to both the T and AT groups, DAI was initiated using the impact-acceleration model of Marmarou et al. [14]. Animals in AT group were given 15 mL/kg of ethanol 0.5 h prior to trauma. Animals of the nonethanol control group in our study were given 15 mL/kg of drinking water by gastric administration. These procedures were performed under general anesthesia induced by intraperitoneal administration of chloral hydrate (0.3 mL/kg i.p.), and repeated doses were administered if necessary. After general anesthesia, the scalp of animals was shaved, a midline incision was performed, and the periosteum covering the vertex was reflected. A stainless-steel disk was fixed to the central portion of the skull vault of the rat between the coronal and lambdoid sutures. 

To deliver TBI, animals were placed in a prone position on a 20 cm thick sponge bed. The injury was then delivered by dropping a 500 g weight from a height of 1.8 m onto the steel disk. Rebound impact was prevented by sliding the flexible sponge bed from the tube immediately. Following termination of the procedure, animals were returned to their normal environment and were provided with food and water.

### 2.2. Imaging

Conventional MRI and DTI were performed in all rats. The control group of ten rats was imaged. Five separate parallel groups of five rats per experimental group (A, T, and AT groups) were imaged at 1, 3, 6, 12, and 24 h after injury. Images were obtained using a 1.5T MR imaging system (GE Signa) equipped with high performance gradients. MRI parameters were as follows: T1 weighted images (T1WI) were obtained using TR (repetition period)/spin-echo time (TE) = 1290 ms/23.2 ms, NEX = 2, section thickness = 3 mm with no gap between sections, matrix = 256 × 256, and field of view (FOV) = 12 cm × 12 cm, T2 weighted images (T2WI) were obtained using fast spin echo sequence, TR/TE = 4420 ms/107.9 ms, NEX = 2, section thickness = 3 mm with no gap between sections, matrix = 256 × 256, and FOV = 12 cm × 12 cm and DTI was obtained with single-shot echo planar imaging (EPI) sequence by using 25 diffusion-encoding directions, TR/TE = 6000 ms/107.7 ms, NEX = 2, section thickness = 3 mm with no gap between sections, matrix = 128 × 128, and display field of view (DFOV) = 6 cm × 6 cm; and the *b* value was 1000 s/mm^2^. Body temperature of all rats was maintained throughout the MRI acquisition. 

### 2.3. Data Processing

**Figure 1:**
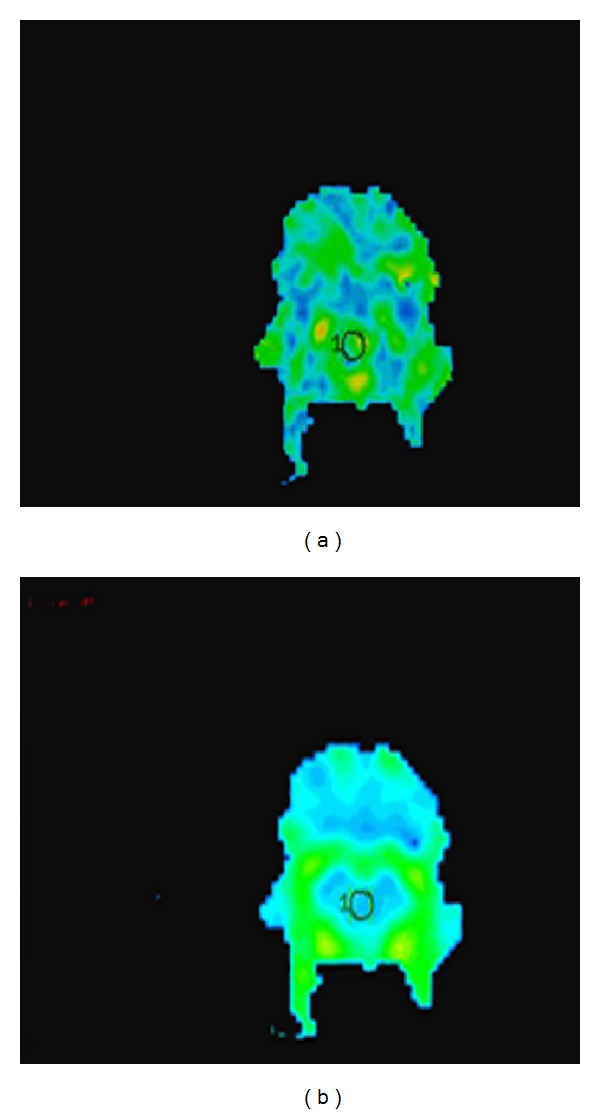
Regions of interest in the rat brainstem. (a) FA map and (b) ADC map.

Images were postprocessed offline by using DTI Studio software (Johns Hopkins University, Baltimore, Maryland) and an Advantage workstation for Windows (AW4.3, GE Healthcare). After correction for movement and EPI-induced distortion artifacts, the diffusion tensor was calculated for each voxel. The final DTI dataset was fed into Functool 4.5.5 software, which automatically computes the FA and ADC maps. The region of interest was about 5 mm^2^ and was traced on the brainstem in the original DTI transverse slice image to avoid the influence of subjective factors (Figure 1). FA and ADC values for the regions of interest of the control group and each time point of the experimental model were recorded. The average value of the data was measured by two experienced radiologists blinded to the animal status.

### 2.4. Histology

After all images were acquired, animals were immediately sacrificed for histology. Animals received an overdose of chloral hydrate and were perfused transcardially with 4% paraformaldehyde in phosphate buffer. Brains were removed and fixed with 4% paraformaldehyde for 24 h. After fixation, brains were embedded in paraffin, and contiguous 5 *μ*m sections at the level of the brainstem were cut on a microtome (Rm 2016, LEICA, Germany). Sections were stained with hematoxylin and eosin (HE) or Bielschowsky's silver stain, and immunohistochemistry of AQP4 was performed. 

### 2.5. AQP4 Immunostaining

After being washed with phosphate-buffered saline (PBS), sections were treated with 0.3% hydrogen peroxide for 10 min to inactivate endogenous peroxidase. After being washed 3 times, 5 min each, with 0.01 M PBS, sections were blocked in 10% goat serum for 10 min at room temperature. Then sections were incubated with ready-to-use rabbit anti-AQP4 (BA-1560, Wuhan, China) overnight at 4°C, followed by a 30 min incubation at 37°C with biotinylated goat anti-rabbit secondary antibody (ZDR-5306, Beijing, China). Visualization was performed by incubating with diaminobeniydine for 5 min. A negative control was also performed by replacing the primary antibody with PBS. Image acquisition was performed using an Olympus digital camera and dedicated software. For AQP4 quantification, two sections at the level of brainstem were examined from each animal. Using an Image-Pro Plus 6.0 microimage analysis system, integral optical density (IOD) was measured in 5 distinct areas, and the average value was calculated. 

### 2.6. Statistical Analysis

ADC and FA values and AQP4 expression were reported as the mean ± standard deviation (*X* ± SD) in each group. For comparisons within each group and between groups, we used Student's *t*-test. Correlations between DTI parameters and APQ4 expression were calculated using the Pearson test. A *P* value < 0.05 was considered significant. Statistical analyses were conducted using SPSS 13.0 software (SPSS, Chicago, IL, USA).

## 3. Results

### 3.1. Conventional MRI Results

**Figure 2:**
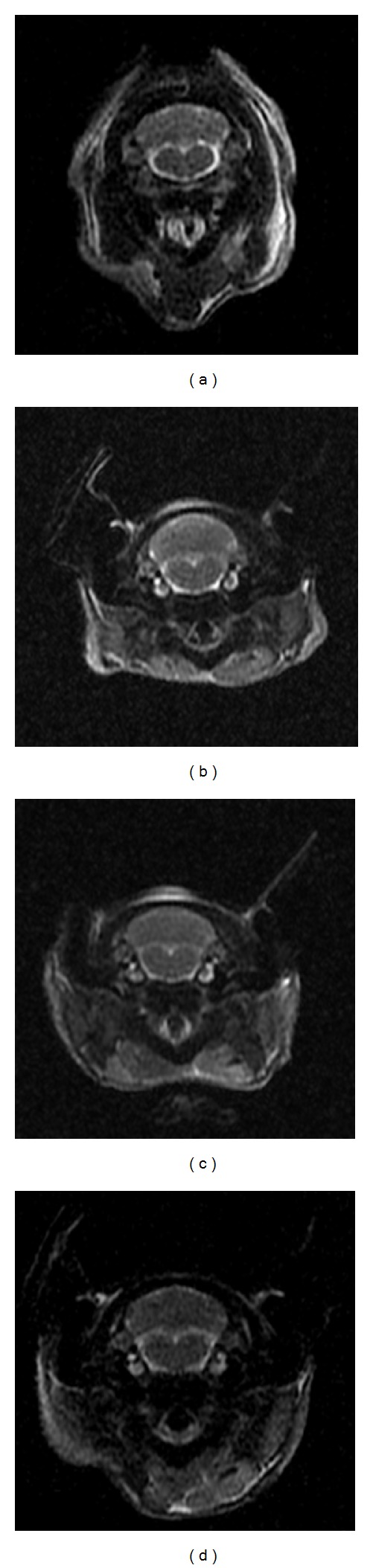
T2WI maps ((a) N group, (b) 6 h in A group, (c) 6 h in T group, and (d) 6 h in T group).

For MRI on control rats, the transverse and sagittal planes of the T1WI and T2WI scans showed clear brain parenchyma structure. Comparison of conventional MR images between control and brain-injured rats, especially T2WI imagings, showed no difference in signal intensity between the N group and each experimental group (Figure 2). These results show that conventional MRI maybe insensitive to detect early neuronal damage following TBI.

### 3.2. DTI Imaging Results

**Figure 3:**
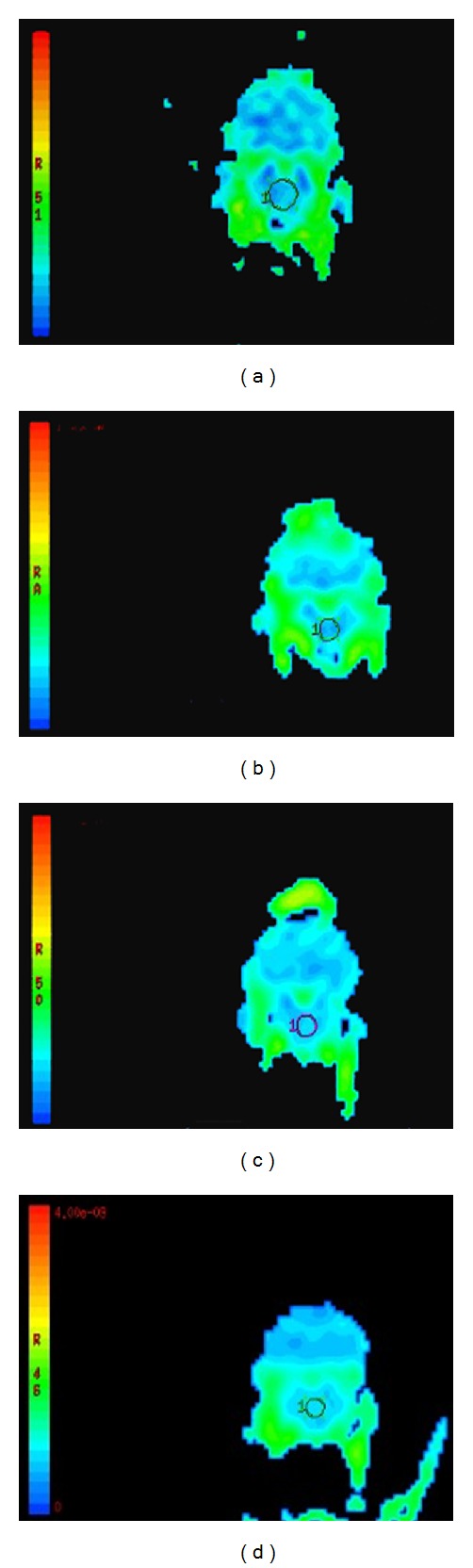
ADC maps ((a) N group, (b) 6 h in A group, (c) 6 h in T group, and (d) 6 h in T group).

**Figure 4:**
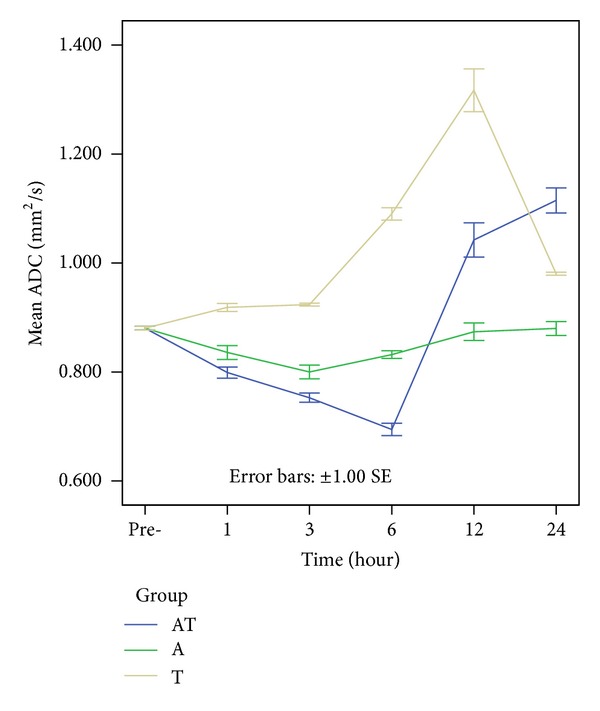
Colored bars represent the mean ADC values of the rat brainstem in the control, A, T, and AT groups. ADCs show a slight reduction within 3 h, followed by a gradual recovery in the A group. ADCs strongly increase, peaking within 12 h, and then decrease in the T group. In the AT group, there is a reduction in ADCs at 6 h after injury, followed by a continued increase at 24 h after injury.

In the control group, the brainstem ADC value was 0.880 ± 0.014 × 10^−3^ mm^2^/s. In the A group, ADC values showed a slight reduction reaching a minimum value at 3 h (0.800 ± 0.056 × 10^−3^ mm^2^/s), indicating the development of cytotoxic brain edema resulting from alcohol administration alone. This was followed by a gradual recovery, reaching a normal ADC value (0.874 ± 0.071 × 10^−3^ mm^2^/s) at 12 h. Compared to the sham-operated control, differences were statistically significant at 1, 3, and 6 h (*P* < 0.05). In the T group, ADC strongly increased relative to the control, suggesting development of vasogenic brain edema. ADC values peaked within 12 h (1.317 ± 0.175 × 10^−3^ mm^2^/s) after DAI, then decreased, but remained higher than the control at 24 h (0.980 ± 0.012 × 10^−3^ mm^2^/s). Differences were significant at 3, 6, 12, and 24 h (*P* < 0.05) compared to the control group. In the AT group, there was a maximal 20% reduction in ADC at 6 h (0.700 ± 0.051 × 10^−3^ mm^2^/s) after injury, followed by a continued increase in ADC to a maximum 31% increase (1.115 ± 0.103 × 10^−3^ mm^2^/s) at 24 h after injury. The decreased ADC posttrauma with acute alcoholism indicates more restricted diffusion, suggestive of cytotoxic brain edema at these time points, whereas the observed increase in ADC after injury suggested the development of vasogenic edema. The difference was statistically significant at all time points compared with the control (*P* < 0.05) (Figures 3 and 4). These results show that DTI could quantitatively measure both ethanol-induced cytotoxic and TBI-induced vasogenic brain edema at early times after injury.

**Figure 5:**
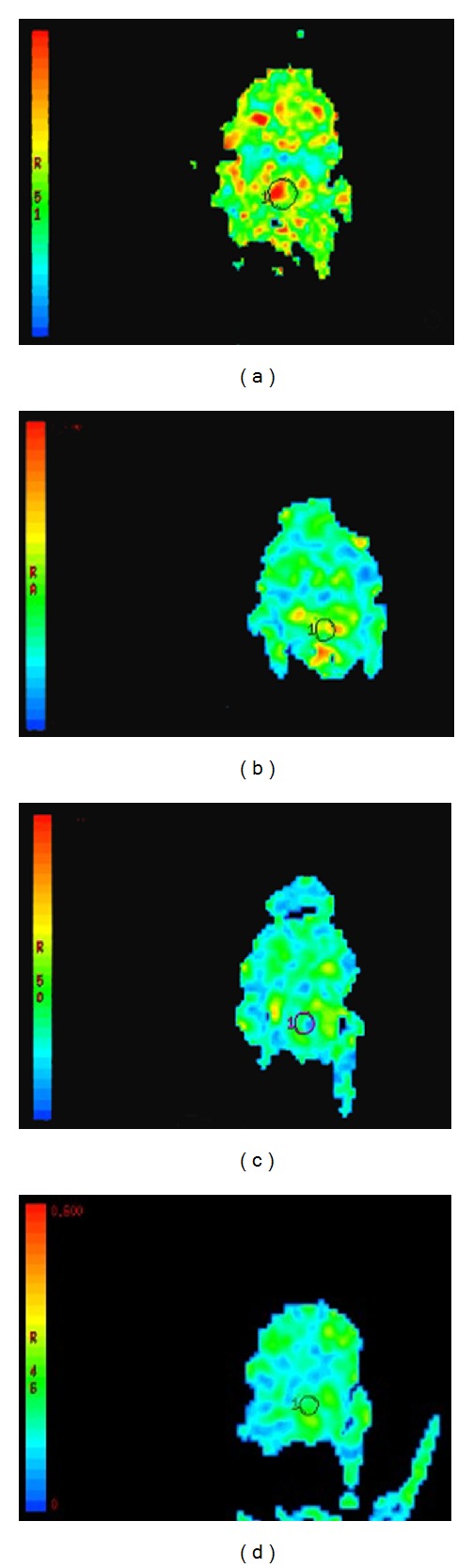
FA maps ((a) N group, (b) 6 h in A group, (c) 6 h in T group, and (d) 6 h in T group).

**Figure 6:**
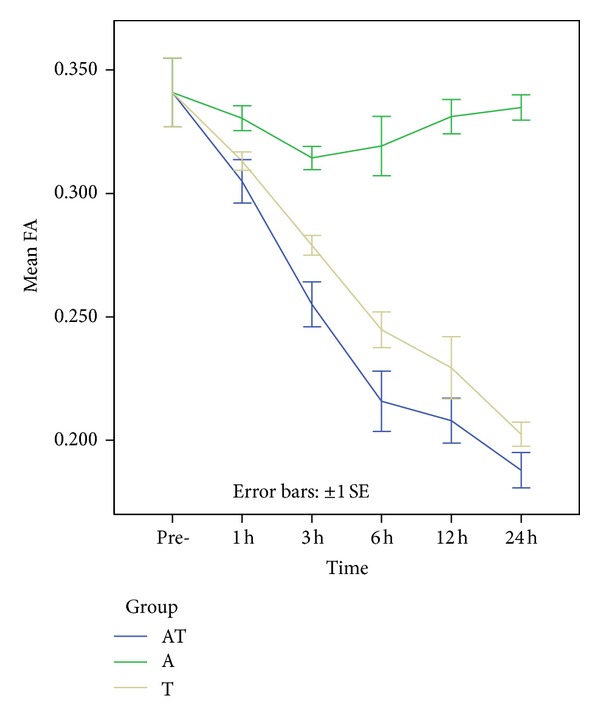
Colored bars represent the mean FA values in the rats brainstem in the control, A, T, and AT groups. No significant difference in the FA values is seen between the control and EtOH treated rats. In the AT and T groups, FA values are continually decreased after injury, reaching a minimum value. At 3 h and 6 h following DAI, FA values are lower in the AT group than in the T group.

In the control group, the brainstem FA value was 0.341 ± 0.062. In the A group, the temporary reduction in FA values was not significantly different from the control. In the AT and T groups, FA values continually decreased after injury, beginning at 60 min after trauma, reaching a minimum value (T group: 0.202 ± 0.021 and AT group: 0.188 ±  0.032) at 24 h after injury (*P* < 0.01), indicating progressive development of axonal injury. FA values were lower in the AT group than in the T group, with differences being significant at 3 h and 6 h after injury (*P* < 0.05), suggesting that alcohol exacerbates the decrease in FA; that is, alcohol enhances diffuse axonal injury (Figures 5 and 6).

### 3.3. Histological Results

#### 3.3.1. HE

**Figure 7:**
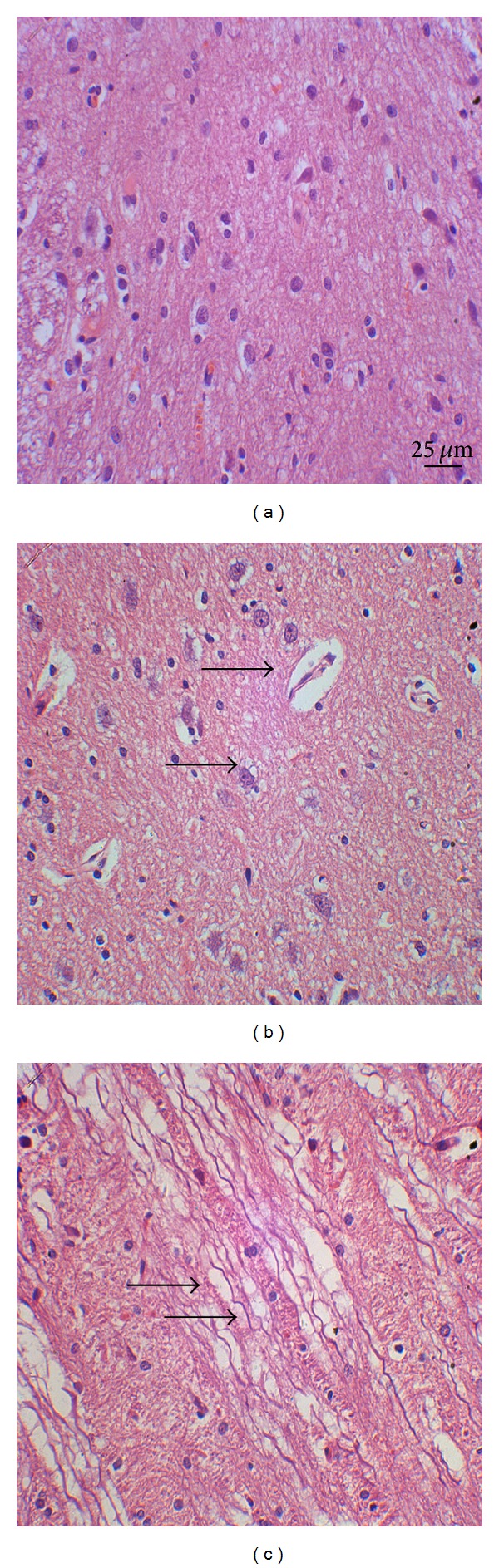
HE stain of the rat brainstem. (a) Control group. (b) 3 h and (c) 12 h after DAI under acute alcohol intoxication show cell swelling. Cell swelling, weakly stained plasma, collapse of blood vessel, shrunken endothelium, and enlarged expanded extracellular space of axons are observed (black arrows).

No pathologic changes in the brainstem were detected in the control rats (Figure 7(a)). After acute alcohol intoxication, the principal corresponding histologic changes within 6 h were intracellular edema, such as cell swelling, weakly stained plasma, and narrowed extracellular space of cells. The T group showed expanded cells, expanded extracellular space of cells, weakly stained plasma, enlarged space of axons, and shrunken endothelium. The AT group showed cell swelling, weakly stained plasma, narrowed and expanded extracellular space, collapse of blood vessel, shrunken endothelium, and enlarged expanded extracellular space of axons (Figures 7(b) and 7(c)). These results suggest that, when compared to results of the T group, these morphological changes corresponding to brain edema were more prominent in the AT group after 6 h.

#### 3.3.2. Bielschowsky's Silver Stain

**Figure 8:**
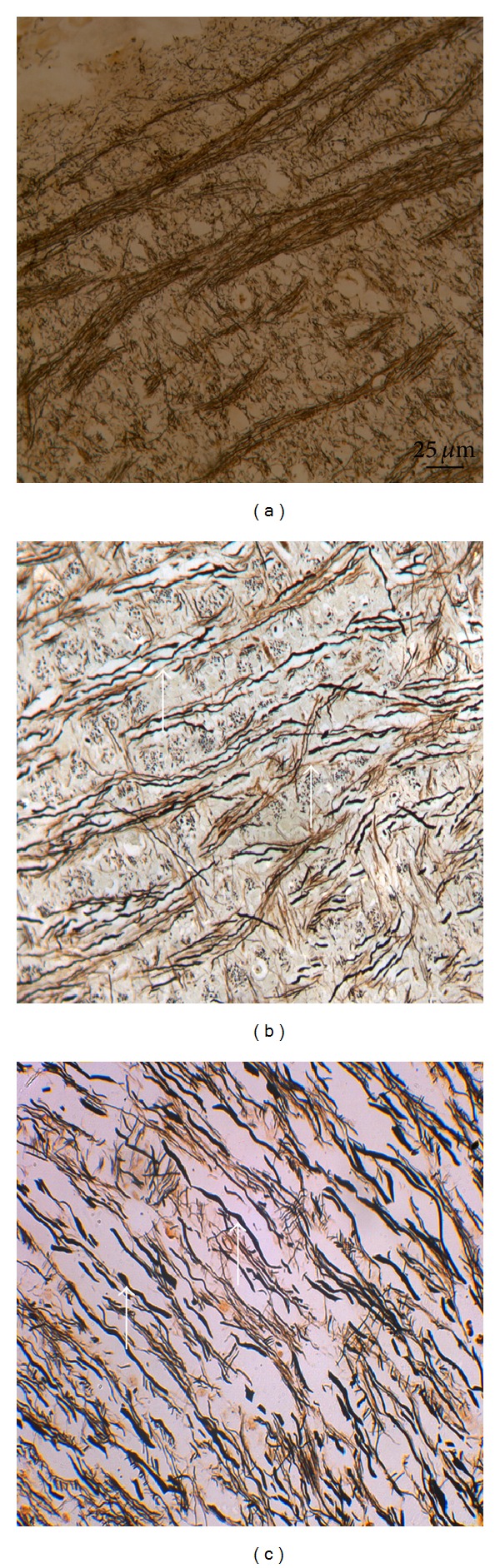
Bielschowsky's silver stain of rat brainstem. (a) Control group. (b) 3 h and (c) 12 h after DAI under acute alcohol intoxication show axon swollen, twisted, disrupted, and enlarged axonal space, and axonal retraction bulbs are observed (white arrows).

Silver stain was performed to detect axonal injury. The axon stains were well distributed, without being twisted or disrupted in control rats (Figure 8(a)). The A group showed axon swelling and slight twisting but no significant disorganization. In the AT group, after brain trauma, the principal corresponding histologic changes were axon swelling and twisting with the axonal space enlarged. Axons became irregular, partly truncated, and retracted. 3 h later, after injury axonal retraction bulbs were generated. As time progressed, the axonal retraction bulbs increased and were most obvious at 24 h (Figures 8(b) and 8(c)). In the T group, axons were swollen, twisted, and partly disorganized, and generated axonal retraction bulbs were observed. These results indicate axonal injury was more pronounced in the AT group compared to the T group.

#### 3.3.3. AQP4 Expression

**Figure 9:**
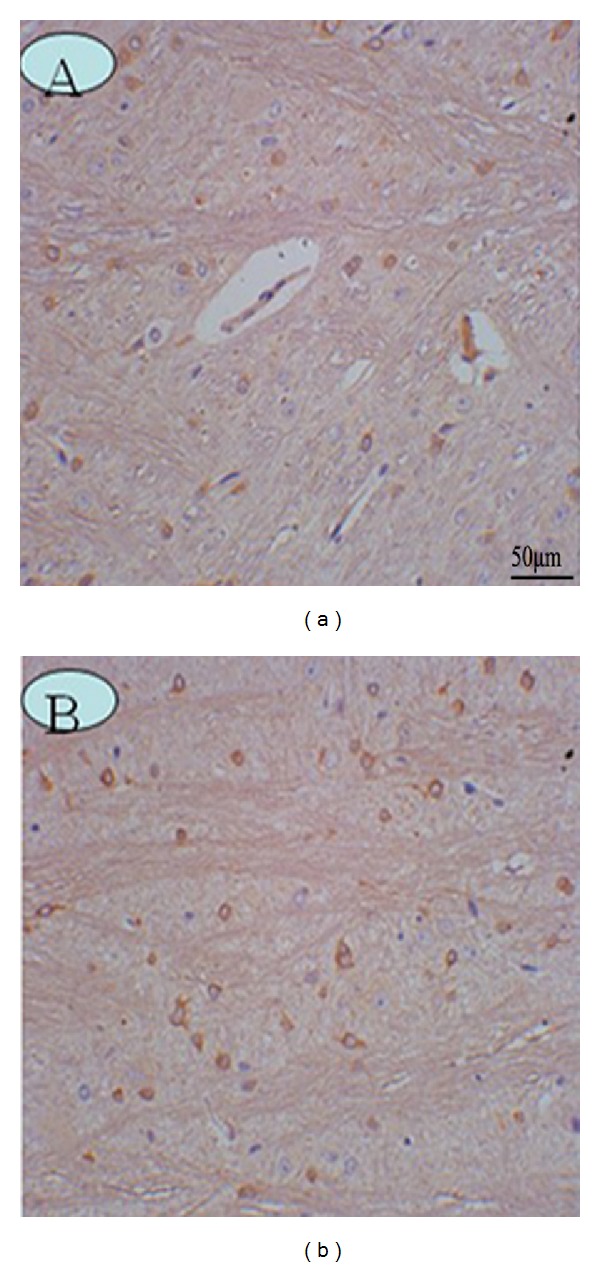
AQP4 expression in the rat brainstem. (a) Control group. (b) 12 h after DAI under acute alcohol intoxication shows AQP4 immunoreactivity is more pronounced along the entire reactive astrocyte membrane.

**Figure 10:**
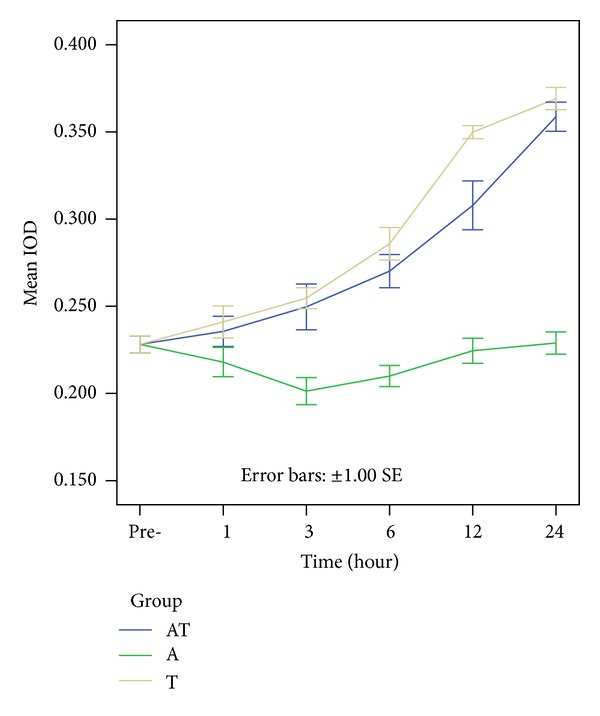
Colored bars represent the mean AQP4 expression in the rat brainstem in the control, A, T, and AT groups. In the A group, there is a slight decrease of AQP4, reaching a minimum at 3 h, then increasing. Both in the T and AT groups, there is moderate increase in brainstem AQP4 expression, reaching a peak at 24 h. At 12 h following trauma, AQP4 are high in the T group than in the AT group.

Activity of AQP4, the predominant aquaporin in the brain, has been implicated in brain edema. We examined changes in AQP4 expression following brain injury. In the control group, the OD of brainstem AQP4 was 0.228 ± 0.021. In the A group, there was a slight decrease in the immunoreactivity of AQP4 compared to the control group, reaching a minimum at 3 h (0.209 ± 0.027, *P* < 0.05), then increasing. In the T group, the AQP4 immunoreactivity was strongly upregulated compared to the control group, reaching a peak at 24 h (0.369 ± 0.028, *P* < 0.01). In the AT group, there was a moderate increase in brainstem AQP4 expression, compared to the control group, which also increased to a peak at 24 h (0.358 ± 0.037, *P* < 0.01). The OD of AQP4 was higher in the T group compared to the AT group, with differences becoming statistically significant at 12 h (*P* < 0.05), indicating that brainstem AQP4 was up-regulated after DAI with acute alcoholism, and that alcohol may inhibit the expression of AQP4 after DAI (Figures 9 and 10).

#### 3.3.4. DTI/Histological Correlations after DAI with Acute Alcoholism

We found negative correlations between ADC values and the brainstem AQP4 expression within 6 h (*r* = −0.532, *P* < 0.01) and positive correlations between 6 h and 24 h (*r* = 0.500, *P* < 0.01) in the AT group. The change in correlation could be reflective of the change in type of edema, from a cytotoxic to a vasogenic form. Negative correlations between FA values and AQP4 expression were observed at 24 h in the AT group (*r* = −0.497, *P* < 0.01). This could reflect axonal damage, such as misalignment of the cytoskeletal network, changes in the axonal cylinder shape, or disconnection of white matter tracts.

## 4. Discussion

DAI involves progressive injury, beginning with local swelling of axons, followed by cytoskeletal perturbations, including misalignment of fibers and eventual disconnection. FA is determined by several factors, including the thickness of the myelin sheath and of the axons as well as the organization of the fibers and properties of the intracellular and extracellular space around the axon. Changes in tissue structure (misalignments of the cytoskeletal network or the axonal membranes permeability) caused by DAI after acute alcohol intoxication can lead to a modification of the degree of directionality, leading to the changes of FA and ADC. It indicated that DTI can probe microscopic structural changes after DAI after acute alcohol intoxication; this finding will contribute new information to the basic science of axonal injury.

In the present study, we used a rats' model for DAI to demonstrate that DTI is capable of detecting early changes in brainstem brain edema and axonal injury under conditions where the brain appears normal under conventional MRI. There are different clinical outcomes between the treatment of traumatic vasogenic brain edema and cytotoxic edema of the same drug, if the evolution of ADC value changes of brainstem over time occurs in human DAI with acute alcoholism patients similar to that described here in our rat model; the finding of development of brain edema is of great importance on effective therapeutic strategies.

Earlier reports showed that acute exposure to EtOH (ethanol) can disturb water balance and induce cellular edema in cerebral tissues [15, 16]. This effect is mainly due to the influx of EtOH across the plasmalemma, driven by its concentration gradient, effectively increasing intracellular osmolarity, and disturbing ion homeostasis [15]. Alcohol can elevate intraneuronal Ca^2+^ and inhibit Na^+^/K^+^ ATPase activity, resulting in intracellular Na^+^ accumulation and eventual cytotoxic edema. TBI also induces mechanical damage, excitotoxic damage, alterations in Ca^+^ homeostasis, and mitochondrial dysfunction, resulting in brain edema [17, 18]. Wilde et al. [12] summarizes one mechanism that involves the potential of alcohol use to alter pathophysiologic responses to injury through a traumatically induced imbalance of neurotransmitter action which may increase excitotoxic reaction. And alcohol may impact hemodynamic and respiratory brainstem control centers accentuating acidosis through longer posttraumatic apnea periods, decreased ventilation, impaired ventilatory responses, subsequently leading to decreased cerebral perfusion pressure, and lower postinjury regional cerebral blood flow for hours after injury. Also, another mechanism implicates alcohol as a factor in facilitating or exacerbating BBB disruption and increased permeability in brain regions close to the site of impact following injury.

Our previous investigation demonstrates that reduced ADCs can allow the diagnosis of cytotoxic brain edema in cases of acute alcohol administration [19]. Calculation of ADC offers real-time detection and differentiation of the type of edema formation after DAI [20]. After DAI with acute alcoholism, reduced ADC values are thought to be secondary to the decrease in the extracellular space caused by cell swelling. In pathological conditions, ADC values represent water movement within tissues and reduced values are thought to be associated with decreases in the extracellular space caused by cell swelling. This interpretation is hypothetical and the underlying physiological basis of the ADC remains incompletely understood [21]. A reduction of diffusion is linked to cytotoxic edema, which results from the failure of the cellular membrane Na^+^/K^+^ pump, and increases in the average spacing between neurons and gliocytes due to vasogenic brain edema resulting from DAI with acute alcoholism. ADC values represented water movement within tissues and increased values are thought to be associated with excess extracellular fluid accumulation. 

ADC values from TBI studies in animals have shown differences in the relative contribution of vasogenic and cytotoxic edema after TBI. Previous ADC results have been more variable following TBI. Zheng et al. found reduced ADC in brainstem, deep gray matter, and corpus callosum, from lesions depicted on diffusion-weighted images, and concluded that this was consistent with cellular edema [22]. High ADC values have been reported at the site of contusion, but reduced or near normal ADC values have been reported in the perilesional brain tissue, tissue distant from the lesion on the ipsilateral side, and tissue in the contralateral hemisphere [23]. 

Our observation of decreased FA provides evidence of brainstem axonal injury after DAI with acute alcoholism, consistent with prior studies using DTI to show reduced FA following TBI [24, 25]. In our studies, the AT group showed lower FA values in the brainstem compared with the control and T group, indicating alcohol may aggravate axonal injury. The observed reduction in anisotropy may reflect misalignment of the cytoskeletal network, changes in the axonal cylinder shape, or disconnection of white matter tracts. Reductions in FA may be the consequence of edema, which may be reversible, or of traumatic axotomy, which is irreversible. Based on DTI/pathological comparisons, we demonstrated that FA decreases are associated with axon swelling and disconnection after trauma. Combining silver stain changes with FA values was helpful for characterizing the development of axonal injury after DAI with acute alcohol intoxication. The negative correlations between FA values and AQP4 expression at 24 h in the AT group may reflect an increase in radial diffusion with misalignment of the cytoskeletal network in DAI, implying a broadening of the diffusion ellipsoid. In the acute phase, this broadening is consistent with axonal swelling, which reflects brain edema to some extent, leading to the upregulation of AQP4 expression after DAI under acute alcoholism. 

Moreover, we found that acute alcohol intoxication may inhibit brainstem AQP4 expression. We show that ethanol alters AQP4 expression following TBI. Upregulation of AQP4 is implicated in the formation and resolution of cerebral edema after DAI under acute alcohol intoxication. Though astrocytes protect the function of neurons, astrocyte AQP4 permits astrocytic absorption of excess water of the neuron during brain edema. The increase of AQP4 in astrocytes leads to continued increase in cell volume due to more water entering the cell, resulting in cell body swelling, causing cerebral edema, and increasing intracranial pressure. It has been suggested that astrocytes may utilize AQP4 to maintain extracellular homeostasis, where excess water may preferentially flow into astrocytes following TBI. AQP4 inhibitors would reduce cytotoxic brain swelling only if administered early to slow the entry of edema fluid into the brain parenchyma. Administering AQP4 inhibitors late after the onset of cytotoxic edema, or in vasogenic edema, is predicted to increase brain swelling. AQP4 facilitates the clearance of extracellular fluid in the brain [26]. Augmentation in AQP4 expression and/or function may, thus, be beneficial in reducing brain swelling in vasogenic edema and in the resolution of phase of cytotoxic edema. Our results show acute alcoholism may inhibit the expression of AQP4, thus, alleviating cytotoxic cerebral edema at early stages of DAI under acute alcohol intoxication. The alcohol-mediated inhibition of AQP4 could aggravate vasogenic brain edema by blocking fluid clearance following vasogenic brain edema that follows mechanical injury with DAI under acute alcohol intoxication.

Our study shows that ADC values are correlated to the level of AQP4 expression under pathological conditions. Decreased ADC values are reflective of cytotoxic edema, which may be due to increased AQP4 expression, and increased ADC values are interpreted as reflective of vasogenic edema, which may be due to increased AQP4 expression. Cytotoxic edema results from disruption of normal osmotic gradients across the plasma membrane, causing an osmotically induced flux of water into cells and a primarily intracellular edema. AQP4 has an important role in the accumulation of intracellular fluid, the expression of AQP4 increase in astrocytes, that leads to the continued increase into the cell volume and more water entering in cell. The up-regulation of AQP4 expression is likely to aggravate ADC reduction. Vasogenic edema develops by an aquaporin-independent mechanism involving increased BBB permeability, resulting in extracellular accumulation of edema fluid. ADC values represented water movement within tissues and increased values are thought to be associated with excess extracellular fluid accumulation secondary to BBB disruption caused by brain injury; AQP4-mediated transcellular water movement is crucial for fluid clearance in vasogenic brain edema [27]. The up-regulation of AQP4 may represent a protective response to facilitate the clearance of excess brain water.

## 5. Conclusion 

In summary, DTI is capable of detecting the effect of acute ethanol administration on diffuse axonal injury; changes of ADC and FA values indicate the effect of ethanol on brain edema and the severity of axonal injury. Ethanol inhibits the expression of AQP4 after acute alcoholism with DAI; AQP4 is upregulated in response to brain edema following DAI with acute alcohol alcoholism. The correlations between ADC and the brainstem AQP4 expression at different time points suggest AQP4 expression follows an adaptative profile to the severity of brain edema.
